# MYO5B Deficiency-Associated Cholestasis and the Role of the Bile Salt Export Pump

**DOI:** 10.3390/cells15010092

**Published:** 2026-01-05

**Authors:** Zhe Zhou, Sven C. D. van IJzendoorn

**Affiliations:** Department of Biomedical Sciences, University of Groningen, University Medical Center Groningen, Antonius Deusinglaan 1, 9713 AV Groningen, The Netherlands; z.zhou@umcg.nl

**Keywords:** familial intrahepatic cholestasis, PFIC, myosin-Vb, BSEP, genotype-phenotype, rare disease, liver, case report, pathogenesis, immunohistochemistry

## Abstract

**Highlights:**

**What are the main findings?**

- Aberrant BSEP localization correlates with the absence of truncating nonsense or frameshift variants in *MYO5B*.
- Clinical phenotypes do not differ between cases with normal versus aberrant BSEP localization.

**What is the implication of the main findings?**

- The clinical picture is more relevant than genotype or immunohistochemistry findings to guide management.

**Abstract:**

Variants of the *MYO5B* gene, which encodes the molecular motor protein myosin-Vb, have gained prominence as a causative factor in familial intrahepatic cholestasis (FIC). Understanding the disease mechanism is pivotal for therapy development and clinical decision-making. The prevailing theory for the mechanism underlying *MYO5B*-associated cholestasis implicates faulty trafficking of the *ABCB11*-encoded bile salt export pump (BSEP) in hepatocytes due to dysfunctional myosin-Vb. This is supported by cell and mouse studies. However, while BSEP localization was abnormal in some patients’ liver biopsies, BSEP appeared normally localized in others, raising questions with regard to the role of BSEP in *MYO5B*-associated FIC. We present a focused systematic narrative review of all cases of *MYO5B* variant-associated isolated FIC reported in the MEDLINE database. We assembled a comprehensive patient dataset and assessed clinical features of *MYO5B*-associated FIC, their relationship with *MYO5B* genotype, the clinical value and significance of BSEP abnormalities, and the relationship of *MYO5B*-associated FIC to *ABCB11* variant-associated FIC. Our review revealed that aberrant BSEP localization correlated with the absence of one *MYO5B* allele carrying a truncating nonsense or frameshift variant. Notably, biochemical and clinical parameters including treatment outcome were indistinguishable between patients presenting with normal and aberrant BSEP localization. Further, myosin-Vb and BSEP deficiency-associated FIC patient cohorts showed distinct biochemical and clinical phenotypes, indicating different underlying mechanisms. This suggests that whether or not BSEP localization was abnormal depended on the *MYO5B* genotype without a predictable effect on clinical parameters and treatment response. Treatment decisions should be guided by clinical parameters rather than by genotype or immunohistochemistry findings.

## 1. An Introduction to MYO5B-Associated Cholestasis and the Role of the Bile Salt Export Pump (BSEP)

Familial intrahepatic cholestasis (FIC) is an inherited severe and debilitating liver disease for which treatment options are limited to off-label symptomatic medical therapies or surgical interventions [1,2]. FIC can present as benign recurrent intrahepatic cholestasis (BRIC) or as progressive familial intrahepatic cholestasis (PFIC). In this article we will use the term FIC. The major types of FIC are caused by pathogenic variations in the *ATP8B1*, *ABCB11* and *ABCB4* genes. These encode the ATPase phospholipid-transporting protein 8B1 (ATP8B1), bile salt export pump (BSEP) and multidrug resistance protein 3 (MDR3), respectively, which all reside at the bile canalicular plasma membrane. With the emergence of affordable genome sequencing, new FIC-associated genes have recently been discovered [1,3,4,5]. Of these, *MYO5B*-associated cholestasis (OMIM#619868) emerges as a considerable group, estimated to represent up to 25% of FIC cases other than those caused by variations in the *ATP8B1*, *ABCB11* and *ABCB4* genes [6].

Despite its prevalence, there has been limited examination of the biochemical and clinical aspects of *MYO5B*-associated cholestasis, and its clinical and mechanistic relationship with other FIC types remains unclear. The *MYO5B* gene encodes myosin-Vb. Myosin-Vb is a motor protein involved in vesicular trafficking of apical plasma membrane proteins via RAB11A-positive recycling endosomes [7]. In the liver, these RAB11A-positive recycling endosomes have been shown to traffic BSEP to the apical bile canalicular plasma membrane of hepatocytes, where it is responsible for intrahepatic bile flow [8]. Previous studies demonstrated that expression of motor-deficient C-terminal tail fragments of myosin-Vb with RAB11A-binding capacity impaired vesicular trafficking of BSEP and other bile canalicular transporters in hepatocyte cell lines [8,9]. Aberrant myosin-Vb and BSEP immunohistochemistry (IHC) results have been observed in liver biopsies of *MYO5B*-associated cholestatic patients [6,10]. In liver organoids from a cholestatic patient, correction of the *MYO5B* variant resulted in a restoration of the BSEP-mediated Tauro-nor-THCA-24-DBD transport [11]. Further, expression of a human *MYO5B* variant in the mouse liver causes cholestasis and aberrant BSEP localization in the hepatocytes [12]. Supported by these observations, the pathological mechanism of *MYO5B*-associated cholestasis has been attributed to aberrant BSEP trafficking. It is conceivable that myosin-Vb deficiency may impact the expression and trafficking of other proteins and transporters, including MDR3, ABCG5/G8 or the water channel aquaporin-8, but such correlation has not been published so far. It has been proposed that immunostaining for BSEP and other markers can differentiate various FIC subtypes including *MYO5B*-associated FIC [13,14].

However, in other case reports of *MYO5B*-associated cholestasis, BSEP IHC results showed normal BSEP expression at the bile canalicular membrane [15,16,17], which may challenge this proposed mechanism. The aim of this article was to provide a focused systematic narrative review of all cases of *MYO5B* variant-associated isolated FIC reported in the MEDLINE database, assemble a comprehensive patient dataset and assess clinical features of *MYO5B*-associated FIC, their relationship with *MYO5B* genotype, the clinical significance of BSEP abnormalities, and the relationship of *MYO5B*-associated FIC to BSEP variant-associated FIC.

## 2. Clinical Characteristics of MYO5B-Associated Cholestasis

### 2.1. Case Report Inclusion

**Figure 1:**
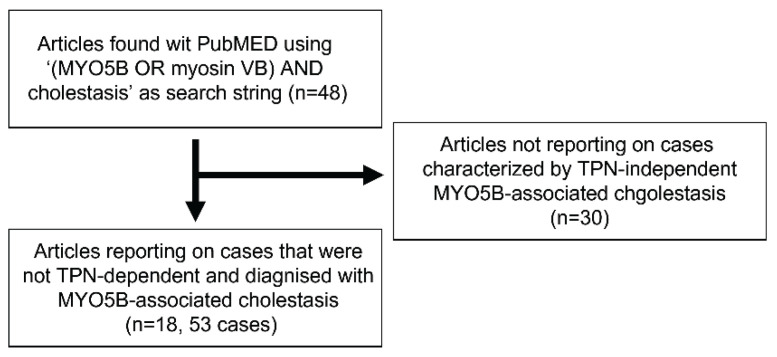
Flowchart of case inclusion from PubMed search (date accession: 22 May 2025). Flowchart of case inclusion from MEDLINE search (date accession: 22 May 2025). TPN: total parenteral nutrition.

All published reports were retrieved from the MEDLINE database using the search string “((MYO5B OR myosin-Vb) AND cholestasis)”. Unique hits were screened for reports of cases that were diagnosed with *MYO5B*-associated cholestasis. Total parenteral nutrition (TPN) dependency (typically cases of *MYO5B* variant-associated microvillus inclusion disease (MVID)) was an exclusion criterion as BSEP expression may be affected by long-term TPN. Data was retrieved from 53 patients reported in 18 studies (Figure 1; Supplemental Table S1).

### 2.2. Symptoms and Liver Biochemistry

A variety of data (including clinical data, genetics, serum biochemistry, biopsy inspections, treatment and treatment outcomes) was extracted from the 53 case reports (Supplemental Table S1). Data availability differed between patients and a data availability chart is provided in Supplemental Figure S1. Patients included 37 males and 15 females, indicating a male preponderance (odds ratio = 0.4, *p* = 0.02). The median onset of symptoms was 6.5 months (IQR 2.75–11) with one outlier (i.e., higher than Q3 + 1.5 × IQR or lower than Q1 − 1.5 × IQR) of 180 months. Symptoms were pruritus (84%), jaundice (45%), hepatomegaly (38%) and splenomegaly (10%). The severity of pruritus was rarely reported in a measurable manner, for example by the numerical or verbal rating scales or visual analog scale of pruritus. Further, reports of chronic, intermittent or transient pruritus often did not correlate with the narrative description of severity in those cases, which altogether hinders the careful assessment of symptom severity.

**Figure 2:**
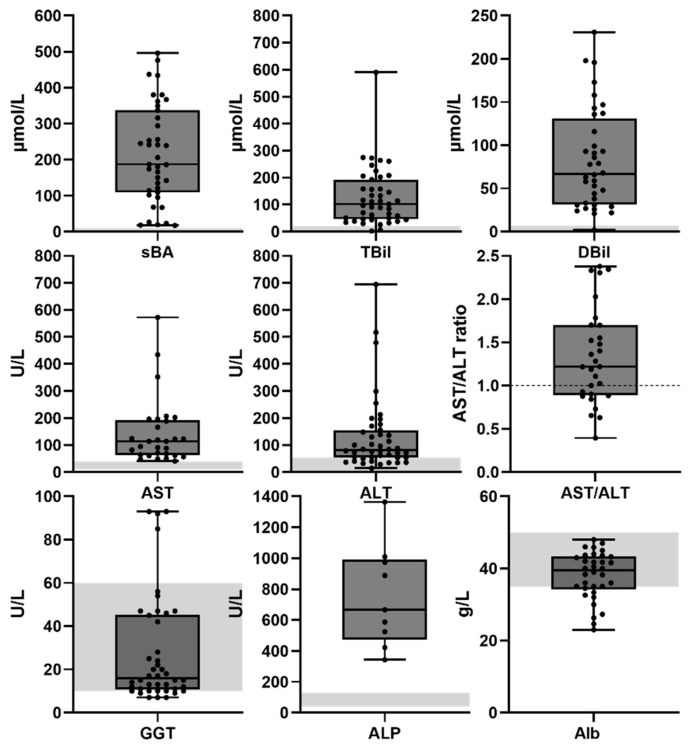
Serum biochemistry *MYO5B*-associated cholestasis. Serum biochemistry data are presented as box plots with individual data points and median. Gray area indicates normal ranges. AST: aspartate aminotransferase; ALT: alanine aminotransferase; TBil: total serum bilirubin; DBil: direct bilirubin; sBA: serum bile acids; GGT: gamma glutamyltransferase; ALP: serum alkaline phosphatase; Alb: serum albumin.

**Figure 3:**
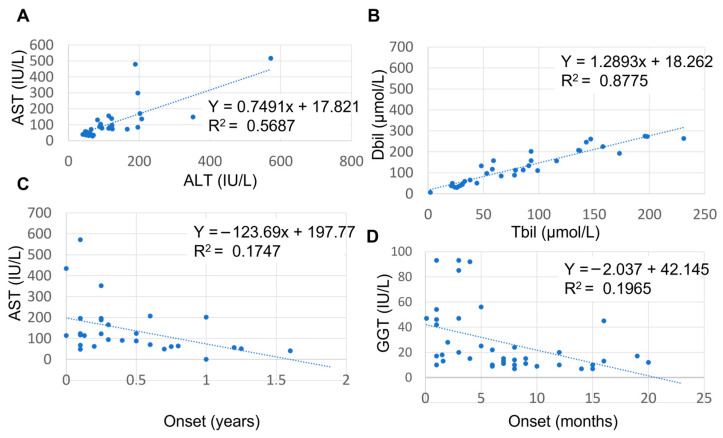
Relationship between AST and ALT, AST/ALT ratio and age, and between GGT and age. (**A**) Positive relationship between AST and ALT; (**B**) negative correlation between DBil and TBil; (**C**) negative relationship between AST and age of onset. (**D**) negative correlation between GGT and age of onset. AST: aspartate aminotransferase; ALT: alanine aminotransferase; GGT: gamma glutamyltransferase.

At the group level, increased serum levels were observed for alanine aminotransferase (ALT) (median 82 international units (IU)/L (interquartile range (IQR) 54.4–163 with three outliers), aspartate aminotransferase (AST) (median 114 IU/L (IQR 63–192.5) with 2 outliers, direct bilirubin (DBil; median 67 µmol/L (IQR 31.5–131)), total bilirubin (TBil; median 102 µmol/L (IQR 47–200) with 1 outlier), percentage direct bilirubin (median 66% (36–89)) and serum bile acids (sBa; median 187 µmol/L (IQR 96.75–310.5)). Serum gamma-glutamyltransferase (GGT) (median 16 IU/L (IQR 10.75–45.25)) and serum albumin (median 39.5 g/L (IQR 34.5–35.4)) were within normal ranges (Figure 2). Prothrombin time and serum alkaline phosphatase (ALP) were elevated in all cases but not consistently reported (only in 8 and 18% of cases, respectively) and not included in further analyses. Total serum protein was within normal range but only reported in 10% of all cases and not included in further analyses. Nonetheless, the reported prothrombin time, ALP and serum protein values are included in Supplemental Table S1. Strong per-patient positive correlations were observed between AST and ALT values (Pearson correlation; *r*(27) = 0.745, *p* < 0.0001) (Figure 3A) and between DBil and TBil values (Pearson correlation; *r*(34) = 0.936, *p* < 0.001) (Figure 3B). No correlations were found between other combinations of ALT, AST, GGT, sBA and TBil values (Pearson correlations; ALT vs. TBil: *p* = 0.82, ALT vs. sBA: *p* = 0.24, sBA vs. TBil: *p* = 0.96) (Supplemental Figure S2). The lack of correlation of transaminases with bilirubin or bile acids suggests that these enzyme changes do not reliably predict cholestasis and underscores the need for a multi-marker assessment when evaluating liver dysfunction in these patients.

### 2.3. Age-Related Serum Biochemistry Findings

Notably, the levels of AST and GGT but not of ALT, sBa, DBil or TBil were age dependent. When we separated cases with a reported onset of <6 months of age from those with an onset > 6 months of age (Supplemental Table S1), AST values were elevated with a large variation in cases under 6 months of age, whereas in cases above 6 months of age, AST values were within the normal range (Figure 3C). When comparing AST and ALT values and AST/ALT ratio variance between these two age groups we found these to be statistically significant (F-test; *p* = 0.03). While the median GGT value was within normal range, individual GGT values were occasionally elevated to almost 100 U/mL (Supplemental Table S1). GGT values and age of onset showed a moderate to strong negative correlation (Figure 3D; Pearson correlation; *r*(38) = −0.44, *p* = 0.004). The higher GGT values likely reflected the patients’ younger age, which is a known determinant of serum GGT levels in the healthy pediatric population.

### 2.4. Treatment and Treatment Response

From the data accumulated in Supplemental Table S1, we found that treatment with ursodeoxycholic acid (UDCA) was reported in 86% of cases. Treatment with cholestyramine and rifampicin was reported in 44 and 47% of cases, respectively. Of the cases with pruritus, 50% were treated with both cholestyramine and rifampicin and 26% were treated with cholestyramine or rifampicin. Treatment with fat-soluble vitamins was reported in 27% of cases. Other drug treatments included phenobarbital and antihistamines. Surgery to reroute bile salt flow was performed in 11% of cases, three of which were in the same medical center. Two patients (4% of cases) received a liver transplant; one patient died while listed for a liver transplantation. There was a statistically significant correlation between surgical biliary diversion and the onset of liver symptoms at a later age (median 15 versus 6.5 months for patients subjected to surgery or not, respectively; Kruskal–Wallis test *p* = 0.002). With regard to pruritus treatment response, 22% of cases responded well to the treatment received, and 22% cases did not respond to treatment. Mild or temporary relief of symptoms (i.e., recurrent or intermittent pruritus or cholestasis) was reported in 56% of cases. By comparing patients from Supplemental Table S1 for whom information was available with regard to treatments and symptom recovery, we found that recovery of symptoms was not dependent on surgical biliary diversion versus pharmacological treatment (Fisher exact test; *p* = 0.691; Supplemental Figure S3A).

## 3. Genetic Characteristics of MYO5B-Associated Cholestasis

### 3.1. Types and Combinations of MYO5B Variants

**Figure 4:**
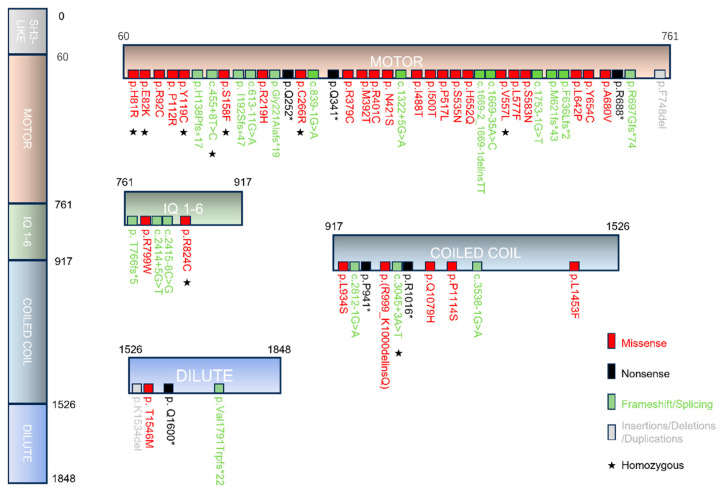
Schematic representation of myosin-Vb variants in *MYO5B*-associated cholestasis. Scheme showing the myosin-Vb protein, its domains, and the location of known homozygous (indicated by asterisks), heterozygous and compound heterozygous mutations (missense in red, nonsense in black, insertions/deletions/duplications in gray and frameshift/splicing variants in green text color). Protein data are deduced from GenBank RefSeq-file accession number NG_012925.1 for the human *MYO5B* gene. Nucleotide numbering reflects cDNA numbering with +1 corresponding to the A of the ATG translation initiation codon in the reference sequence. The initiation codon is codon 1.

None of the patients carried biallelic nonsense or frameshift variants predicted to give rise to a premature termination codon (PMT), in agreement with earlier reports [6,10,18,19]. The majority of patients with *MYO5B*-associated cholestasis carried at least one missense variant (78%) or biallelic missense variants (47%) in the *MYO5B* gene. Twenty-eight distinct missense variants of *MYO5B* were reported in cholestatic patients (Figure 4). The majority of the missense variants located to the myosin-Vb motor domain, while intra-protein amino acid interaction network analyses showed no variant hotspots (Supplemental Figure S4A). Homozygosity was reported in 22% (10/45) of cases.

### 3.2. Myosin-Vb-p.(Arg824Cys) and -p.(Arg92Cys)

We found that approximately one-third of patients with *MYO5B*-associated cholestasis carried the p.(Arg824Cys) and/or p.(Arg92Cys) variant. The p.(Arg824Cys) variant caused cholestasis when overexpressed in *Myo5b*-knockout mouse livers [12]. Neither variant has been reported in patients diagnosed with *MYO5B*-associated MVID (https://www.mvid-central.org; accessed on 16 December 2025). Hence, the p.(Arg824Cys) and p.(Arg92Cys) variants are highly prevalent and specific for *MYO5B*-associated cholestasis.

Arg824 is highly conserved and located at the third alpha-helical isoleucine–glutamine (IQ) domain ([I,L,V]QxxxRGxxx[R,K]) of myosin-Vb. Based on the AlphaFold-predicted structure of full-length human myosin-Vb (AF-Q9ULV0-F1-v6), Arg824 interacts with Gln820. Arg820 regulates the interaction of myosin-V with calmodulin. By using the SWISS-MODEL server (https://swissmodel.expasy.org/, accessed on 25 November 2025) to model the Arg824-to-Cys824 substitution (see legend to Supplemental Figure S4A), we found that the Arg824Cys substitution is predicted to eliminate the hydrogen bond with Gln820, in agreement with the loss of the H-bond-donating side chain of Arg when substituted by Cys. This possibly affects calmodulin binding and myosin activity regulation. p.(Arg824Cys) and p.(Arg92Cys) are not involved in interactions with other amino acids known to be mutated in *MYO5B*-associated cholestasis (Supplemental Figure S4A). Based on the AlphaFold-predicted structure of full-length human myosin-Vb (AF-Q9ULV0-F1-v6), Arg92 is located in the motor domain and is predicted to form hydrogen bonds with residues of the transducer domain (Supplemental Figure S4B). This domain modulates the kinetics and stability of the different myosin V states during the actin–myosin cycle. To determine how the Ar92Cys variant may affect myosin Vb in the different states, we used the SWISS-MODEL server and the crystal structure-based structures of chicken myosin V in different nucleotide states to model the Arg92-to-Cys92 substitution, we find that a cysteine at the homologous position is predicted to eliminate these hydrogen bonds in the nucleotide-bound but not nucleotide-free state of the myosin cycle (Supplemental Figure S4B). This possibly affects the efficiency and coordination of the motor. While these in silico predictions warrant experimental validation, both variants have been shown to display impaired motor activity in an in-cell assay [20]. By comparing patients from Supplemental Table S1 for who information was available with regard to their genetic variants and treatment outcomes, we found that the presence or absence of the p.(Arg824Cys) and p.(Arg92Cys) myosin-Vb variants did not correlate with laboratory results or with treatment outcome (Fisher exact test; *p* = 0.06 and 0.49, respectively; Supplemental Figure S3B,C).

## 4. The Role of BSEP in MYO5B-Associated Cholestasis

### 4.1. BSEP Expression in MYO5B-Associated Cholestasis: A Genotype–Phenotype Relationship

The mechanism underlying *MYO5B*-associated cholestasis has been attributed to abnormal localization of BSEP in liver cells, essentially mimicking *ABCB11* deficiency-associated FIC [6,8,9,12]. We assessed BSEP immunohistochemistry (IHC) in liver tissue from 16 *MYO5B*-associated cholestatic patients reported in seven studies (Supplemental Table S2). All studies included one or more positive control liver tissues, which displayed the typical canalicular staining pattern of BSEP. Several cases of *MYO5B*-associated cholestasis showed a normal canalicular BSEP staining pattern, while other cases displayed an abnormal pattern (Table 1) described as “reduced staining”, “intracellular” or “sub-canalicular” staining. Aberrant expression or localization of BSEP was observed in less than half of all cases for which BSEP IHC was reported.

By comparing patients from Supplemental Table S1 for whom information was available with regard to their genetic variants and BSEP IHC results, we found that the likelihood of observing abnormal BSEP IHC was linked to whether one of the alleles contained a nonsense or frameshift *MYO5B* variant (Table 1; Fisher exact test; *p* = 0.04). Specifically, 75% of cases displaying normal BSEP distribution had one of these specific variants, resulting in the production of the mutant myosin-Vb from a single allele. In contrast, 88% of cases exhibiting abnormal BSEP distribution had compound heterozygous or homozygous missense mutations in *MYO5B*, which do not necessarily lead to a loss of myosin-Vb protein. This correlation demonstrated a sensitivity (true-positive rate) of 88% and a selectivity (true-negative rate) of 75%.

This suggests a threshold hypothesis in which the amount of the mutant myosin-Vb protein, determined by the number and type of mutated alleles, influenced the presence or absence of BSEP abnormalities. When only one allele was involved in producing myosin-Vb variants the impact on BSEP IHC may not be visually evident. However, when both alleles contributed to myosin-Vb variants, this was adequate to affect BSEP.

Three cases (Supplemental Table S1) deviated from this genotype–phenotype relationship. A patient with compound heterozygous p.(Phe748del)/p.(Ile577Phe) variants and another with the homozygous p.(Arg824Cys) variant showed normal BSEP distribution, suggesting that these variants, expressed from two alleles, were not disruptive enough or were expressed at too-low protein levels to affect BSEP expression. In another patient, monoallelic expression of the myosin-Vb-p.(Arg401Cys) variant (in the absence of protein from the other allele) was sufficient to affect BSEP expression.

While the proposed threshold hypothesis remains speculative and more cases are needed to confirm or refute this correlation, the observed correlation is in agreement with clinical and pre-clinical studies showing that the expression of a mutant myosin-Vb—rather than the loss of myosin-Vb protein—causes an abnormal localization of canalicular proteins via a gain of toxic function [5,8].

### 4.2. BSEP IHC Results Do Not Correlate with Biochemical and Clinical Parameters

In *ABCB11* deficiency-associated FIC, severe or mild *ABCB11* variants have been associated with distinct BSEP IHC results and biochemical and clinical characteristics, as well as with treatment response [21,22,23]. One would expect that if abnormal localization of BSEP is part of the mechanism underlying *MYO5B*-associated cholestasis, BSEP IHC results and the *MYO5B* genetic variations might similarly be linked to their clinical and biochemical disease features. However, we found no significant correlations between abnormal BSEP IHC results and measurable disease parameters, including serum bile acids (Table 1). By comparing patients from Supplemental Table S1 for whom information was available with regard to their reported treatment outcomes and BSEP IHC results, we found that the outcome of BSEP IHC also did not associate with narrative descriptions of disease severity in the case reports or with reported treatment outcome (Fisher exact test; *p* = 1; Supplemental Figure S3D).

Because the likelihood of abnormal BSEP IHC results depended on whether one allele carried a nonsense or frameshift *MYO5B* variant, we compared disease parameters between cases with either one allele carrying a nonsense/frameshift variant and one allele carrying a missense variant (i.e., *MYO5B*-Mis/Tru), and cases with both alleles having missense variants (i.e., *MYO5B*-Mis/Mis). Apart from serum bile acids, which had a median value 18% higher in the *MYO5B*-Mis/Tru group (correlated with normal BSEP IHC), there were no significant differences between these groups (Table 1). Hence, the presence or absence of abnormal BSEP IHC results in *MYO5B*-associated cholestasis did not have clinical significance, and the aberrant localization of BSEP clearly was not the primary or sole mechanism responsible for *MYO5B*-associated cholestasis.

### 4.3. Comparison of Clinical Parameters Between MYO5B-Associated Cholestasis and ABCB11 Deficiency-Associated FIC

If a deficiency in BSEP were the major cause of *MYO5B*-associated cholestasis, we would expect the clinical parameters of *MYO5B*-associated and *ABCB11* deficiency-associated cholestasis to be similar. *ABCB11* deficiency-associated cholestasis can be sub-categorized based on mutation severity: FIC1/1 (p.Asp482Gly or p.Glu297Gly on both alleles), FIC1/3 (p.Asp482Gly or p.Glu297Gly on one allele; predicted protein-truncating mutation on second allele) and FIC3/3 (predicted protein-truncating mutation on both alleles). We compared the serum biochemistry and clinical data of *MYO5B*-associated cholestasis with data from the *ABCB11* deficiency-associated cholestasis database (NAPPED) [24,25] (Table 2). *MYO5B*-associated cholestasis differed significantly from FIC(1/1) with regard to age of onset and serum bile acids, from FIC(1/3) with regard to age of onset, ALT and serum bile acids, and from FIC(3/3) with regard to ALT and GGT. The short median follow-up time of patients with *MYO5B*-associated cholestasis (3.35 months; due to the only recent discovery of *MYO5B*-associated cholestasis) prohibited long-term native liver survival analyses. Nonetheless, only 6% of *MYO5B*-associated cholestasis cases underwent liver transplantation before the age of five years, less than reported for *ABCB11* deficiency-associated cholestasis (FIC1/1 10%, FIC3/3 60% and FIC1/3 85%) [21]. None of the *MYO5B*-associated cholestasis cases with a follow-up of >5 years (median 9.1 years) received a liver transplant.

Together, disease parameters from the *MYO5B*-associated and *ABCB11* deficiency-associated cholestasis cohorts displayed significant differences, indicating a potentially distinct underlying cause.

## 5. Conclusions and Perspectives

This review assembled and reviewed a comprehensive literature-derived dataset to generate novel insights and hypotheses with regard to *MYO5B*-associated cholestasis, its relationship with *MYO5B* genotype, the diagnostic and prognostic value and clinical significance of BSEP abnormalities, and the relationship of *MYO5B*-associated cholestasis to *ABCB11*-associated FIC.

This review revealed a possible correlation between the *MYO5B* genotype and the presence or absence of abnormal BSEP IHC results in liver specimens. It also indicated that the presence or absence of aberrant BSEP IHC results did not appear to translate into differences in biochemical or clinical phenotypes or treatment outcome. Supported by the distinctions in biochemical and clinical phenotypes between *MYO5B*-associated and *ABCB11* deficiency-associated cholestasis, this suggests that BSEP IHC on patient biopsies as such does not support MYO5B-associated cholestasis and the pathogenic mechanism underlying *MYO5B*-associated cholestasis reaches beyond BSEP.

Conceivably, endogenously expressed patient-specific variants of *MYO5B*, acting as a key regulator of intracellular trafficking, might have resulted in a mild and partial disruption in the trafficking and expression of various bile canalicular proteins simultaneously. These may have included, in addition to BSEP, FIC-associated ATP8B1 and MDR3, or other canalicular proteins involved in bile homeostasis such as aquaporin-8 [26]. In such a scenario, the combined partially impaired trafficking of multiple proteins, though not individually significant, could collectively have resulted in the clinical presentation of *MYO5B*-associated cholestasis. The reported partially abnormal MDR3 IHC results in the absence of elevated GGT levels in two patients with *MYO5B*-associated cholestasis [9] and the presence of ductular proliferation (typical for *ABCB4*-associated but not *ATP8B1*- or *ABCB11*-associated FIC) in other patients with *MYO5B*-associated cholestasis [6] provide support for this hypothesis. Alternatively, *MYO5B*-associated cholestasis involves other mechanisms such as impaired bile canalicular network development or organization [8,9] or functional interactions between *MYO5B* and other FIC-associated genes and proteins [1,3,5]. Such scenarios imply that molecular therapeutic strategies may be more successful when aimed at the *MYO5B*/myosin-Vb variant rather than its downstream targets.

Our findings warrant a revisiting of our current BSEP-centered view on the pathophysiology of *MYO5B*-associated cholestasis and show that BSEP IHC, unlike recently proposed [13], has thus far held no diagnostic or prognostic value for patients with *MYO5B*-associated cholestasis. Treatment decisions should therefore be guided by clinical parameters rather than by genotype or immunohistochemistry findings.

The following notes of caution apply. Firstly, whether obtained tissue and IHC images accurately represented the entire liver could not be established. Secondly, not all case reports mentioned whether patients were screened for *ABCB11* polymorphisms, which may have affected the BSEP-IHC outcome. Also, UDCA treatment might have affected the relationship between genotype and BSEP IHC results [27]. However, we found no correlation between UDCA treatment and BSEP-IHC outcomes. Thirdly, differences in drug treatment regimen and doses (not typically reported) may have influenced treatment outcome. We assumed international guidelines on drug treatment were followed. Fourthly, the study had some missing data due to its retrospective design. However, these were minimal for the parameters used in the analyses, random and therefore unlikely to have biased the results. In fact, the percentage of cases for which specific data was reported was for many data significantly higher than the reporting rate of other published FIC cohorts (c.f., Table 2). Lastly, we cannot exclude a reporting bias of unusual cases with unexpected findings, which may affect generalization of the validity of the study to the whole *MYO5B*-associated cholestasis patient population. Prospective cohort studies will be instrumental in testing the hypotheses generated in this review.

## Figures and Tables

**Table 1:** Comparison of cases with normal and aberrant BSEP IHC results and between cases with MYO5B-Mis/Tru and -Mis/Mis variants.

	*MYO5B*-Associated Cholestasis BSEP-IHC Normal Median [IQR]; (*n* = 8)	*MYO5B*-Associated Cholestasis BSEP-IHC Aberrant Median [IQR]; (*n* = 8)	*p* Value
1 ns/fs *MYO5B* variant (*n*, %)	6/8 (75)	1/8 (13)	0.04
0 ns/fs *MYO5B* variant (*n*, %)	2/8 (25)	7/8 (88)	0.04
Patient/disease parameters			
Females (*n*, %)	2/8 (25)	2/8 (25)	1
Onset of symptoms (months)	7 [3–12]	4.5 [1–10]	0.19
Available (%)	8 (100)	8 (100)	
ALT (IU/L)	139 [43.75–186.25]	88 [62–170]	0.87
Available (%)	4 (50)	7 (88)	
GGT (IU/L)	15 [9.5–52.3]	11 [10–47]	1
Available (%)	5 (63)	7 (88)	
sBA (µmol/L)	136.5 [43.75–275]	223 [163.5–287.75]	0.23
Available (%)	4 (50)	7 (88)	
	*MYO5B*-Associated Cholestasis (*MYO5B*-Mis/Tru) Median [IQR]; (*n* = 14)	*MYO5B*-Associated Cholestasis (*MYO5B*-Mis/Mis) Median [IQR]; (*n* = 21)	*p* Value
Females (*n*, %)	6/14 (43)	5/20 (25)	0.46
Onset of symptoms (months)	3 [1–11.25]	7 [4.5–13]	0.23
Available (%)	14 (100)	21 (100)	
ALT (IU/L)	86.5 [51.5–132.25]	86.3 [63.5–181.75]	0.48
Available (%)	10 (71)	18 (86)	
GGT (IU/L)	20 [14.5–33.5]	12.5 [9.75–45.5]	0.33
Available (%)	9 (64)	18 (86)	
sBA (µmol/L)	221.5 [82.5–324.25]	186 [26–362]	0.002
Available (%)	6 (43)	19 (90)	
DBil (µmol/L)	48 [31–93]	66 [29–99]	0.80
Available (%)	7 (50)	16 (76)	
TBil (µmol/L)	70 [39.5–174]	104 [40.25–117.75]	0.71
Available (%)	9 (64)	16 (76)	
Treatment/treatment outcome			
UDCA (*n*, %)	12/14 (86)	16/21 (76)	0.68
Cholestyramine (*n*, %)	7/14 (50)	8/21 (38)	0.51
Rifampicin (*n*, %)	10/14 (71)	10/21 (48)	0.29
Surgery (*n*, %)	2/14 (14)	3/21 (14)	1
LTx (*n*, %)	1/14 (7)	1/21 (5)	1
Responsive to treatment	3/14 (23)	2/21 (11)	0.62
Nonresponsive to treatment	4/14 (31)	2/21 (11)	0.21
Partially responsive to treatment	6/14 (46)	14/21 (78)	0.13

Continuous data were expressed as medians and interquartile range. Non-parametric Mann–Whitney and Kruskal–Wallis tests were used were applicable. Categorical data were expressed as *n* (%) and analyzed using the Fisher exact test. Correlations between two continuous variables were determined using Pearson correlation tests. A two-sided *p*-value < 0.05 was considered statistically significant. Abbreviations and ranges: TBil, total bilirubin (5.0–17.1 µmol/L); DBil, direct bilirubin (0–6 µmol/L); ALT, alanine transaminase (0–40 IU/L); GGT, gamma-glutamyltransferase (7–50 IU/L); sBA, total bile acids (0–10 µmol/L); UDCA, ursodeoxycholic acid; LTx, liver transplantation. ns/fs, nonsense/frameshift; IHC, immunohistochemistry; IQR, interquartile range.

**Table 2:** Comparison of disease parameters between MYO5B-associated and *ABCB11* deficiency-associated cholestasis.

	*MYO5B*-Associated Cholestasis (*n* = 52)	*ABCB11* Deficiency-Associated Cholestasis FIC2(1/1) ^1^ (*n* = 31)	*p* Value *MYO5B* vs. *ABCB11* FIC2(1/1)	*ABCB11* Deficiency-Associated Cholestasis FIC2(1/3) ^1^ (*n* = 30)	*p* Value *MYO5B* vs. *ABCB11* FIC2(1/3)	*ABCB11* Deficiency-Associated Cholestasis FIC2(3/3) ^1^ (*n* = 77)	*p* Value *MYO5B* vs. *ABCB11* FIC2(3/3)
Females (n, %)	15 (29)	16 (52)	0.06	19 (63)	0.01	40 (52)	0.02
Onset of symptoms (years)	0.55 [0–15]	0.8 [0.3–1.9]	<0.001	1.3 [0.5–4.4]	<0.001	0.7 [0.3–1.9]	0.46
Available (%)	49 (100)	30 (97)	0.14	30 (100)	1	77 (100)	1
ALT (IU/L)	84 [64.5–163]	126 [63–251]	0.12	148 [92–437]	<0.001	293 [138–502]	<0.001
Available (%)	41 (84)	28 (90)	0.51	18 (60)	0.03	64 (83)	1
GGT (IU/L)	16 [10.75–45.25]	15 [10–29]	0.75	22 [18–35]	0.15	27 [18–38]	0.03
Available (%)	41 (84)	26 (84)	1	18 (60)	0.03	65 (84)	1
sBA (µmol/L)	183 [96.75–310.5]	247 [153–378]	0.04	459 [354–529]	<0.001	209 [151–309]	0.24
Available (%)	36 (73)	18 (58)	0.15	11 (37)	<0.001	44 (57)	0.002
TBil (µmol/L)	102 [47–199.75]	95 [44–180]	0.75	110 [57–150]	1	104 [53–145]	1
Available (%)	41 (84)	26 (84)	1	20 (67)	0.10	67 (87)	0.6

^1^: The data in this column were collected from [21]. One-sample sign tests were performed in Excel (version 2108). Formula to calculate the low and high critical values: =BINOM.INV(x,0.5,0.025) and BINOM.INV(x,0.5,0.975), respectively, where x is the total number of values (trials) below and above the H_0_ median. Formula to calculate the associated *p*-value: =BINOM.DIST(x,y,0.5,1), where x is the test statistic (i.e., the minimum of the number of values (trials) that are either above or below the H_0_ median) and y is the total number of values (trials) above and below the H_0_ median. Abbreviations and ranges: TBil, total bilirubin (5.0–17.1 µmol/L); ALT, alanine transaminase (0–40 IU/L); GGT, gamma-glutamyltransferase (7–50 IU/L); sBA, total bile acids (0–10 µmol/L); IU: international units.

## Data Availability

No new data were created or analyzed in this study.

## Supplementary Materials

The following supporting information can be downloaded at: https://www.mdpi.com/article/10.3390/cells15010092/s1, Figure S1: A Data availability chart detailing the types of data and the number of patients from which these data are available. Figure S2: Correlation plots showing the absence of significant correlations between TBil and ALT, sBA and ALT and between TBil and sBA. Figure S3: 2 × 2 and 2 × 3 contingency tables used to calculate Fisher exact tests. Figure S4: Mutated amino acids in myosin Vb amino acid and their interactions with other amino acids within the 3D folded myosin Vb protein; Table S1: Overview of all selected *MYO5B*-associated cholestatic liver disease case reports; Table S2: Mutation details and evaluation of BSEP IHC results of patients with *MYO5B* variant-associated cholestasis.

## Abbreviations

PFIC	progressive familial intrahepatic cholestasis
IHC	immunohistochemistry
BSEP	Bile salt export pump
TPN	Total parental nutrition
sBA	serum bile acids
TBil	total serum bilirubin
DBil	direct serum bilirubin
ALT	alanine aminotransferase
AST	aspartate aminotransferase
GGT	gamma-glutamyltransferase
ALP	Serum alkaline phosphatase
UDCA	ursodeoxycholic acid
MVID	microvillus inclusion disease
IQR	interquartile range
PMT	premature termination codon
NAPPED	NAtural course and Prognosis of PFIC and Effect of biliary Diversion
ATP8B1	adenosine triphosphatase phospholipid transporting 8B1
MDR3	multidrug transporter protein 3
OMIM	Online Mendelian Inheritance in Man
