# Supplementary material for: MYO5B Deficiency-Associated Cholestasis and the Role of the Bile Salt Export Pump
Source: Cells. 2026 Jan 5;15(1):92. doi: 10.3390/cells15010092 (PMC12786198; doi:10.3390/cells15010092)
Supplement: Supplementary file 1 [file cells-15-00092-s001.zip › cells-4027592-supplementary/PFIC10-_supplFigs_R.pdf]

Zhe and van IJzendoorn - MYO5B Deficiency-Associated Cholestasis and the Role of the Bile Salt Export Pump

Supplemental Figures and Tables

Supplemental Figure S1

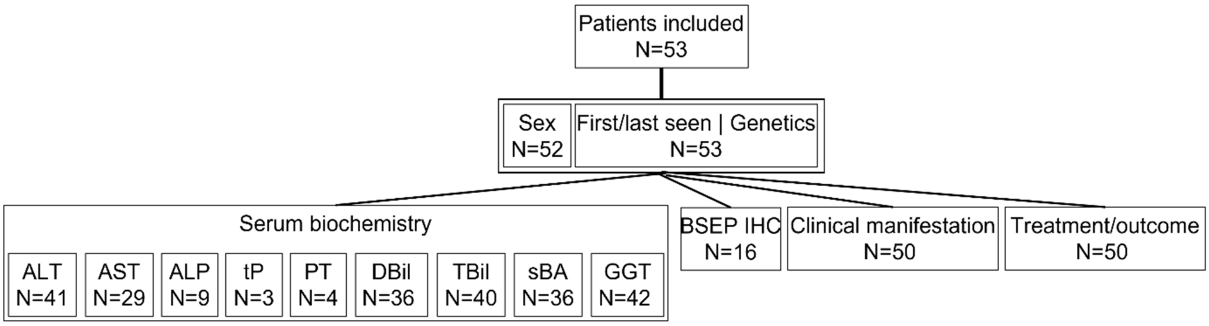

A Data availability chart detailing the types of data and the number of patients from which these data are available.

Supplemental Figure S2

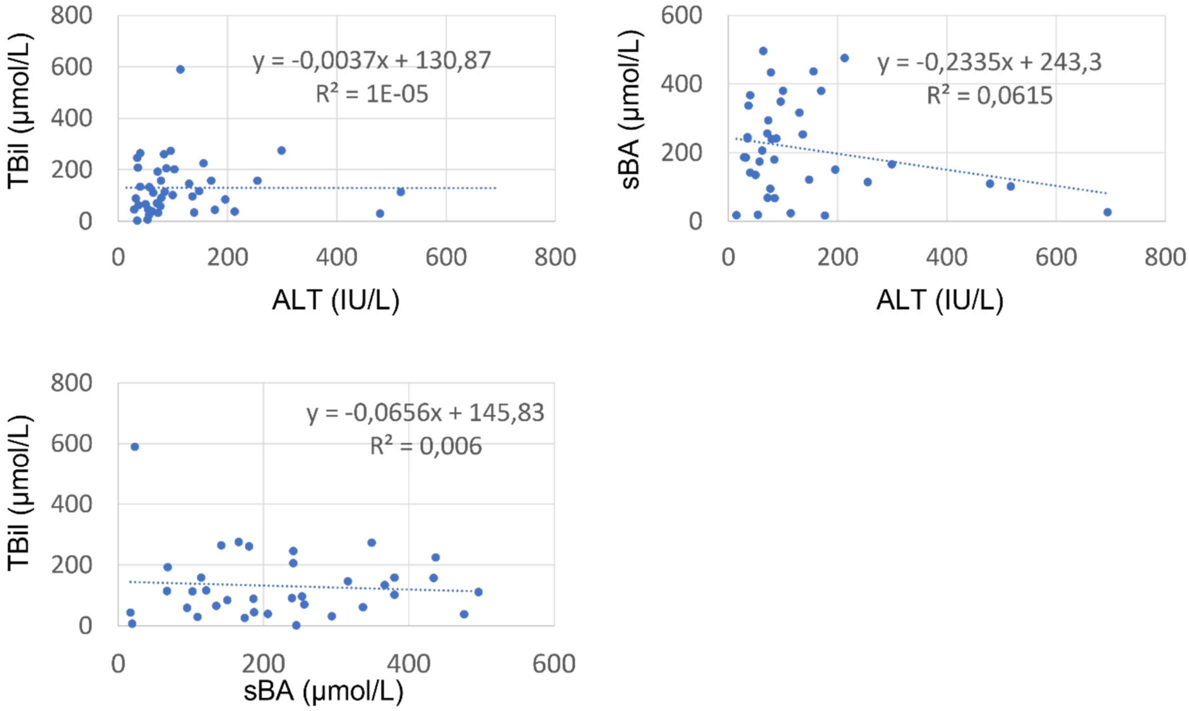

Correlation plots showing the absence of significant correlations between TBil and ALT, sBA and ALT and between TBil and sBA.

Supplemental Figure S3

A

Clinical outcome	Pharm + surggical biliary diversion (n=5)	Pharm - Surgical biliary diversion (n=42)	*P*
Good (n=12)	1	11	0.691
Poor (n=9)	0	9	
Partial/ temporary (n=26)	4	22	

B

Clinical outcome	Arg824Cys (n=16)	No Arg824Cys (n=38)	*P*
Good (n=12)	0	12	0.06
Poor (n=16)	10	6	
Partial/ temporary (n=26	6	20	

C

Clinical outcome	Arg92Cys (n=5)	No Arg92Cys (n=42)	*P*
Good (n=12)	2	10	0.49
Poor (n=10)	0	10	
Partial/ temporary (n=25)	3	22	

D

Clinical outcome	BSEP IHC normal (n=8)	BSEP IHC aberrant (n=7)	*P*
Good (n=3)	2	1	1
Poor (n=1)	0	1	
Partial/ temporary (n=11)	6	5	

2x2 and 2x3 contingency tables used to calculate Fisher exact tests (including Freeman-Halton extension for 2x3 contingency tables).

Supplemental Figure S4

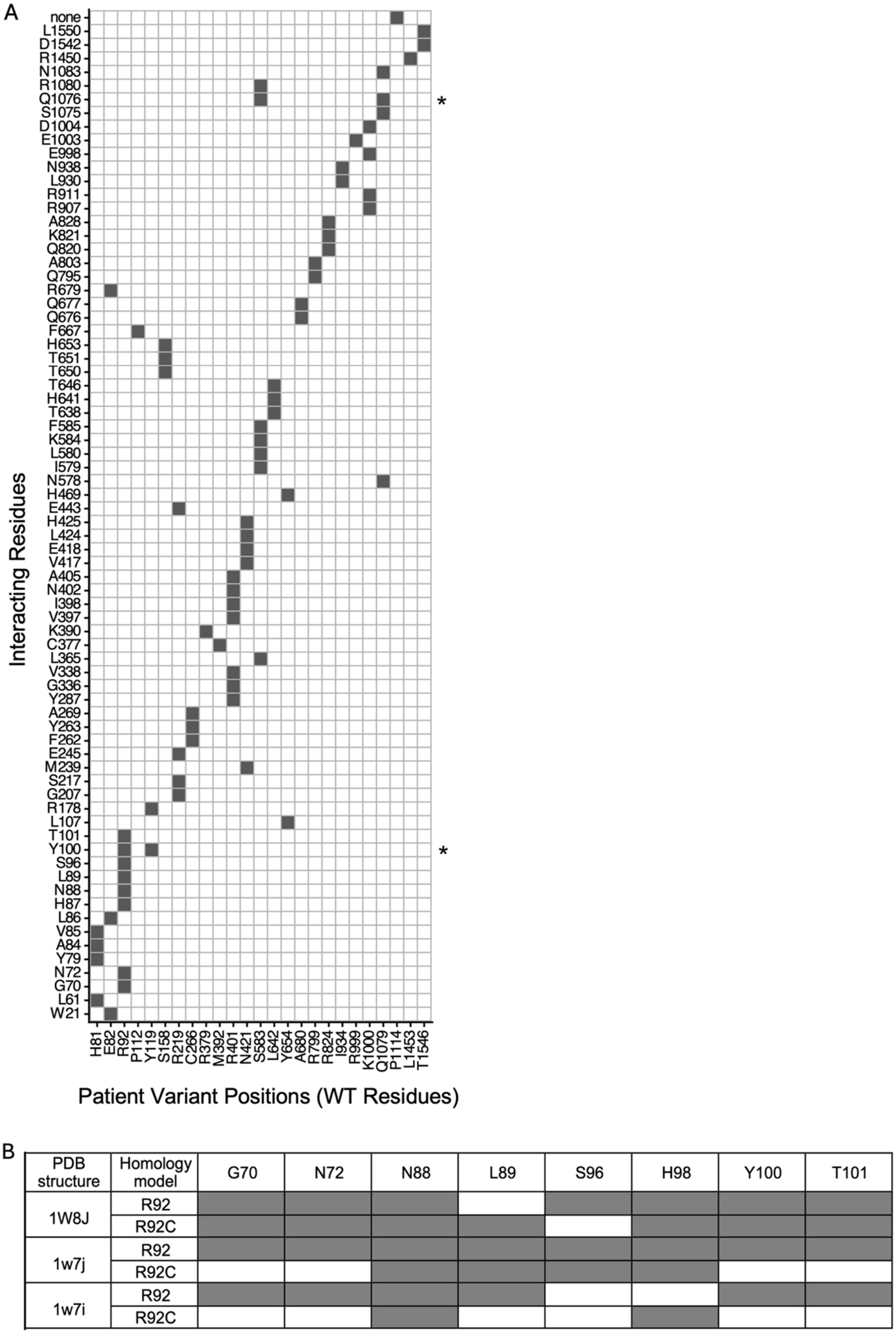

Mutated amino acids in myosin Vb amino acid and their interactions with other amino acids within the 3D folded myosin Vb protein. **A)** Wildtype amino acid residues that have been reported to be altered by missense variants in patients (see Figure 4) are shown on the horizontal axis. The residues they may interact with (through hydrogen bonds) are listed on the vertical axis. Interacting residues on the vertical axis were manually identified from the predicted 3D structure of full-length myosin Vb. For this, The predicted 3D structure of full-length human myosin-Vb (UniProt Q9ULV0; Alpha Fold model AF-Q9ULV0-F1-v6) was downloaded from the AlphaFold Protein Structure Database in PDB format. Intramolecular interactions were identified using PyMOL (Schrödinger, LLC). We focused on residues in myosin Vb that have been reported to be substituted in patients. The residue of interest was first selected via the sequence viewer. Then, neighboring residues within a 5 Å radius were identified to capture local structural context and were displayed in stick representation against a cartoon protein backbone. Polar contact detection in PyMOL was used to identify potential hydrogen bonds based on geometric criteria between polar atoms. Grey boxes indicate potential hydrogen bonds between vertically and horizontally depicted residues within the 3D myosin Vb protein. The asterisks indicate the vertically depicted residues that interact with more than one of the horizontally depicted residues.

B) The SWISS-MODEL server (https://swissmodel.expasy.org/) and the crystal structure-based 3D structure of chicken myosin V in different nucleotide states were used to model the Arg92-to-Cys92 substitution. The amino acids interacting with homologous Arg92 and Cys92 within the 3D crystal structures of the nucleotide-free rigor-like state (1W8J), the MgADP.BeFx-bound post-rigor state (1W7J), and the MgADP-bound rigor state (1W7I) of chicken myosin V are depicted horizontally. Grey and white boxes indicate interaction or no interaction, respectively, with horizontally depicted amino acids within the 3D structure myosin protein.

Supplemental Table S2. Mutation details and evaluation of BSEP IHC results of patients with *MYO5B* variant-associated cholestasis.

Reference **(PMID ID)**	Pt	mutation	BSEP IHC results	Antibody used	IHC quality control assurance parameters	Control included in study	BSEP IHC evaluation
Matarazzo et al., 2022[1] **(35129155)**	5	p.(Ile192Sfs*47) p.(Arg92Cys)	Normal; canalicular	NBP1-89319, Rabbit polyclonal anti-human **BSEP**, (note: same as HPA019035)	Validation by IHC-P; orthogonal validation. Immunogen sequence known (aa 616-756, 4th cytoplasmic domain)	Unspecified control liver tissue	Normal
Matarazzo et al., 2022[1] **(35129155)**	2	p.(Pro517Leu) p.(GlnQ252*)	Normal; canalicular	Same as above	Same as above	Same as above	Normal
Vij et al., 2022[2] **(34338607)**	1	p.(Phe748del) p.(Ile577Phe)	Normal; canalicular	HPA019035, Rabbit polyclonal anti-human **BSEP**	Validation by IHC-P; orthogonal validation. Immunogen sequence known (aa 616-756, 4th cytoplasmic domain)	no	Normal; no control but in agreement with consensus
Cockar et al., 2020[3] **(32304554)**	5	p.(Arg1016*) p.(Met392Thr)	Normal; canalicular	HPA019035, Rabbit polyclonal anti-human **BSEP**	Validation by IHC-P; orthogonal validation. Immunogen sequence known (aa 616-756, 4th cytoplasmic domain)	no	normal; no control hut in agreement with consensus
Cockar et al., 2020[3] **(32304554)**	3	p.(Arg824Cys)	Normal; canalicular	HPA019035, Rabbit polyclonal anti-human **BSEP**	Validation by IHC-P; orthogonal validation. Immunogen sequence known (aa 616-756, 4th cytoplasmic domain)	no	Data not shown
Cockar et al., 2020[3] **(32304554)**	1	p.(Ser158Phe)	Aberrant localization to cytoplasm	sc-74500 Monoclonal anti-human **BSEP**. Santa Cruz.	Validation by Western blot and IHC-P (also in PubMed ID 28027573).	Unspecified control liver	Aberrant localization to puncta in cytoplasm;

					Immunogen sequence known (aa 1-180)		
Qiu et al., 2017**[4]** **(28027573)**	3	p.(Arg401Cys) p.(Gln341*)	Aberrant; reduced staining	sc-74500 Monoclonal anti-human **BSEP**. Santa Cruz.	Validation by Western blot and IHC-P. Immunogen sequence known (aa 1-180). Negative IHC-P control is provided in this study (PFIC2 liver tissue)	incidentally resected normal liver control (adjoining excised tumor); biliary atresia control with cholestasis; confirmed PFIC2 patient with biallelic *ABCB11* mutations (p.I498T / p.R415X); discarded normal liver control (healthy liver donor).	Aberrant; reduced staining;
Qiu et al., 2017**[4]** **(28027573)**	4	p.(Gln1079His) p.(Ser535Asn)	Aberrant; reduced staining	Same as above	Same as above	Same as above	Aberrant; reduced staining; No evidence of aberrant subcellular localization.

Qiu et al., 2017**[4]** **(28027573)**	5	p.(Cys266Arg)	Aberrant; blurred at canaliculi and adjacent cytoplasm	Same as above	Same as above	Same as above	Aberrant
Qiu et al., 2017**[4]** **(28027573)**	6	p.(Ser583Asn) p.(Ile934Ser)	Aberrant; reduced staining	Same as above	Same as above	Same as above	Aberrant; reduced staining;
Qiu et al., 2017**[4]** **(28027573)**	7	p.(Arg697Gfs*74) splicing	Aberrant; reduced staining	Same as above	Same as above	Same as above	Normal when compared to inter/intra-control variation
Gonzales et al., 2017**[5]** **(27532546)**	2	p.(Ile500Thr) p.(Ile642Pro)	Aberrant; thickened canalicular staining and a granular and patchy pattern in the sub-canalicular area	Rabbit anti-rat **BSEP**: gift from Prof. Stieger	Non-commercial Validation by Western blot and IHC-P.	Unspecified control liver	No aberrant localization but dilated BC; good but unspecified control
Gonzales et al., 2017**[5]** **(27532546)**	3	p.(Tyr119Cys)	Aberrant; thickened canalicular staining and a granular and	Same as above	Same as above	Same as above	No aberrant localization but dilated BC; good but unspecified control

			patchy pattern in the sub-canalicular area				
Hess et al., 2021[6] **(33924896)**	1	p.(His81Arg) p.(Gln1600*)	Normal; canalicular	sc-74500 Monoclonal anti-human **BSEP**. Santa Cruz.	Validation by Western blot and IHC-P (also in PubMed ID 28027573). Immunogen sequence known (aa 1-180)	Unspecified control liver	Normal
Hess et al., 2021[6] **(33924896)**	2	p.(Val557Leu)	Aberrant; Additional sub-canalicular localization	Same as above	Same as above	Same as above	Aberrant; Additional sub-canalicular localization

* 1) negative controls, 2) positive controls and 3) antibody validation (3A: immunogen/epitope analysis, 3B: orthogonal validation, 3C: agreement with consensus staining pattern (*i.e.*, consistent staining pattern with a second antibody targeting another epitope)).

Reference list to supplemental table S2

1. Matarazzo L, Bianco AM, Athanasakis E *et al.* MYO5B Gene Mutations: A Not Negligible Cause of Intrahepatic Cholestasis of Infancy With Normal Gamma-Glutamyl Transferase Phenotype. *J Pediatr Gastroenterol Nutr* 2022;**74**:e115–21.

2. Vij M, Shah V. Compound Heterozygous Myosin 5B (Myo5b) Mutation with Early Onset Progressive Cholestasis and No Intestinal Failure. *Fetal Pediatr Pathol* 2022;**41**:811–7.

3. Cockar I, Foskett P, Strautnieks S *et al.* Mutations in Myosin 5B in Children With Early-onset Cholestasis. *J Pediatr Gastroenterol Nutr* 2020;**71**:184–8.

4. Qiu Y-L, Gong J-Y, Feng J-Y *et al.* Defects in myosin VB are associated with a spectrum of previously undiagnosed low γ-glutamyltransferase cholestasis. *Hepatol Baltim Md* 2017;**65**:1655–69.

5. Gonzales E, Taylor SA, Davit-Spraul A *et al.* MYO5B mutations cause cholestasis with normal serum gamma-glutamyl transferase activity in children without microvillous inclusion disease. *Hepatol Baltim Md* 2017;**65**:164–73.

6. Hess MW, Krainer IM, Filipek PA *et al.* Advanced Microscopy for Liver and Gut Ultrastructural Pathology in Patients with MVID and PFIC Caused by MYO5B Mutations. *J Clin Med* 2021;**10**:1901.
